# Synthesis and accumulation of amylase-trypsin inhibitors and changes in carbohydrate profile during grain development of bread wheat (*Triticum aestivum* L.)

**DOI:** 10.1186/s12870-021-02886-x

**Published:** 2021-02-24

**Authors:** Lisa Call, Elisabeth Haider, Stefano D’Amico, Elisabeth Reiter, Heinrich Grausgruber

**Affiliations:** 1 Institute of Animal Nutrition and Feeding, AGES – Austrian Agency for Health and Food Safety, Spargelfeldstr. 191, 1220 Vienna, Austria; 2 Department of Food Science and Technology, BOKU - University of Natural Resources and Life Sciences, Vienna, Muthgasse 18, 1190 Vienna, Austria; 3 Department of Crop Sciences, BOKU - University of Natural Resources and Life Sciences, Vienna, Konrad Lorenz-Str. 24, 3430 Tulln an der Donau, Austria

**Keywords:** ATI metabolism, FODMAPs, Grain development, Trypsin inhibition, Wheat sensitivity

## Abstract

**Background:**

Recent studies indicate that amylase-trypsin inhibitors (ATIs) and certain carbohydrates referred to as FODMAPs (fermentable oligo-, di-, monosaccharides and polyols) play an important role in promoting wheat sensitivity. Hitherto, no study has investigated the accumulation of ATIs during the development of the wheat caryopsis. We collected caryopses of common wheat cv. ‘Arnold’ at eight different grain developmental stages to study compositional changes in ATI and FODMAP content.

**Results:**

The harvested caryopses were analysed for their size, protein and carbohydrate concentrations. ATIs were further characterized by MALDI-TOF MS, and their trypsin inhibition was evaluated by an enzymatic assay. The results showed that ATI accumulation started about 1 week after anthesis and subsequently increased steadily until physiological maturity. However, the biological activity of ATIs in terms of enzyme inhibition was not detectable before about 4 weeks after anthesis. Carbohydrate analysis revealed the abundance of short-chain fructans in early stages of grain development, whereas non-water-soluble carbohydrates increased during later developmental stages.

**Conclusions:**

The results provide new insights into the complex metabolisms during grain filling and maturation, with particular emphasis on the ATI content as well as the inhibitory potential towards trypsin. The time lag between ATI accumulation and development of their biological activity is possibly attributed to the assembling of ATIs to dimers and tetramers, which seems to be crucial for their inhibitory potential.

**Supplementary Information:**

The online version contains supplementary material available at 10.1186/s12870-021-02886-x.

## Background

Amylase-trypsin inhibitors (ATIs) are water-soluble cereal proteins, which also feature good solubility in organic solvents such as chloroform-methanol (CM) mixtures [1]. ATIs were identified as causative proteins for non-celiac wheat sensitivity (NCWS) and other wheat-related disorders [2]. The diseases are characterized by both intestinal and extra-intestinal symptoms that are tightly linked to the consumption of wheat and other gluten-containing foods [3, 4]. As there are no clear diagnostic biomarkers for NCWS, a precise number for prevalence is difficult to obtain, but based on the few data available, prevalence is estimated to range from 0.6 to 10% (for review see [5]).

ATIs were found to initiate innate immune responses by directly activating specific pro-inflammatory receptors (i.e. TLR4) in human body cells, leading to severe inflammations and immune reactions [2, 6]. Additionally, ATIs have the potential to inhibit the activity of two important digestive enzymes in the gastrointestinal system, amylase and trypsin, causing a significant impairment of digestion [7]. Due to this inhibitory feature, ATIs and other metabolic proteins are considered crucial for the natural defence mechanism of the plant itself. They are located in the endosperm of cereal seeds, where they defend starch and protein reserves by blocking amylase and trypsin activities of pathogenic fungi or invading pests [1, 8, 9]. ATIs can be classified, according to their degree of aggregation, into monomeric, dimeric and tetrameric forms. These aggregates are stabilized by non-covalent intramolecular interactions, including disulphide bonds and hydrophobic interactions, resulting in a compact 3D-structure responsible for their high resistance against thermal processing and proteolysis [6, 10]. The inhibitory activity of ATIs is strongly dependent on the 3D-structure and the state of aggregation [1].

Besides ATIs, fermentable carbohydrates, so-called FODMAPs (fermentable oligo-, di-, monosaccharides and polyols), have been implicated in NCWS and are suspected to trigger irritable bowel syndrome (IBS) that causes similar, however exclusively intestinal, symptoms [11]. Among FODMAPs, fructans are most abundant in wheat and wheat-based products. Other FODMAPs, such as free fructose and galacto-oligosaccharides, including raffinose, stachyose and verbascose, were also found to be present in wheat, however in negligible amounts [12].

Although recent studies provided valuable information on the proteomics [13–15] and on fructan metabolism of developing cereals [16–18], the metabolism of ATIs during grain filling and maturation has not been investigated thoroughly so far. In the present study, grains of bread wheat (*Triticum aestivum* L.) cv. ‘Arnold’ were harvested at eight grain developmental stages from anthesis to maturity in order to display the changes in ATIs and carbohydrates during grain filling. Understanding ATI and FODMAP synthesis and regulation during grain filling might be of fundamental importance for breeding purposes.

## Results

### Kernel growth

**Fig. 1:**
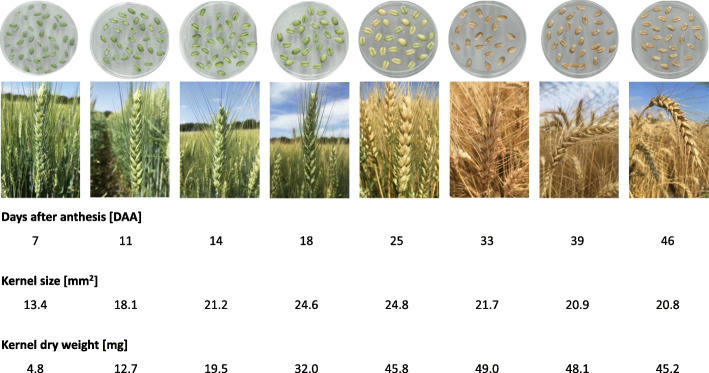
Spike and kernel development in bread wheat cv. ‘Arnold’ during grain filling and maturation. The reported values are mean values obtained from the measurement of at least 20 kernels

Wheat kernel development was studied from anthesis until 46 days after anthesis (DAA) when maturity was reached. Images of developing spikes and kernels as well as their corresponding kernel sizes and kernel dry weights are shown in Fig. 1. While samples taken 7 to 25 DAA showed rather immature ears, spikes and kernels of samples taken 33 to 46 DAA were close to maturity. Caryopses harvested 7 days after full/late flowering (BBCH scale 65–67) were in transition from the watery ripe to early milk stage. Caryopses harvested 18 DAA were in transition from late milk to early dough stage. While samples taken 7 to 18 DAA were still decidedly green, samples harvested 25 DAA were right in the soft dough stage, starting to turn yellow. Ears sampled 33 to 46 DAA had lost all their green color. In general, grain size increased steadily during grain filling and maturation from 13.4 mm^2^ to 20.8 mm^2^ with a maximum of 24.8 mm^2^ at 25 DAA. The slight decrease in kernel size during the later growth stages was corresponding to the final dehydration phase before maturation. Simultaneous to kernel size, dry kernel weight was increasing from 4.8 mg/kernel to 45.2 mg/kernel with a maximum of 49.0 mg/kernel at 33 DAA (Fig. 1 and Additional file 1: Fig. S1). Based on kernel growth analyses and compositional monitoring across the eight sampling dates, grain development could be divided into three phases: cell division and expansion (anthesis to 14 DAA), grain filling (14–25 DAA), grain maturation and desiccation (25–46 DAA) (Additional file 1: Fig. S1).

### Accumulation of proteins during grain development

**Fig. 2:**
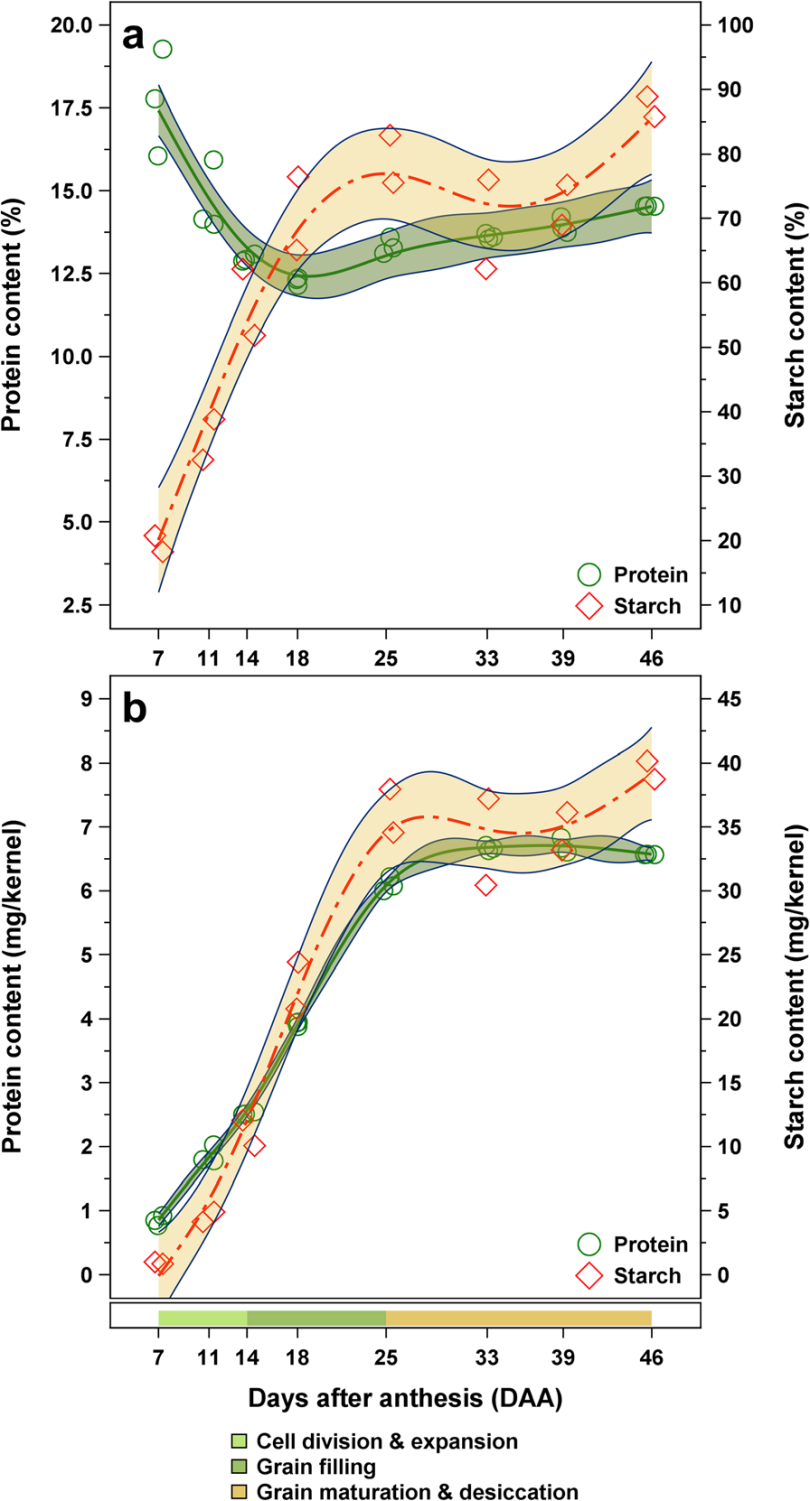
Changes of grain characteristics during seed development of bread wheat cv. ‘Arnold’. **a** Crude protein (green line and circles and 95% confidence interval, *n* = 3 per developmental stage) and starch content (red line and diamonds, *n* = 2) displayed as percentage and **b** mg per kernel, respectively

Protein contents of the developing wheat kernels derived by Dumas and Bradford method as well as RP-HPLC are shown in Table 1 and Fig. 2. RP-HPLC chromatograms displaying the separation of non-gluten proteins including ATIs upon the eight developmental stages can be found in Additional file 2: Fig. S2. In general, crude protein concentration showed considerable variation (12–18%) in the time frame of this study (i.e. 7 to 46 DAA). In the early stages of grain development, crude protein content declined from 17.7 to 12.3% (Fig. 2a), whereas kernel size and dry kernel weight were increasing enormously (Additional file 1: Fig. S1). After reaching a minimum of 12.3%, further protein was accumulated resulting in 14.5% at final maturity. While protein content per 100 g of flour was found to be slightly increasing after an initial decrease (Fig. 2a), protein content on a single kernel basis was steadily increasing in the first phase of grain development until a final protein content of 6.6 mg/kernel was reached (Fig. 2b). Concentrations on a single kernel basis for other traits are listed in Additional file 3: Table S1 and Additional file 4: Table S2. On the contrary, the amounts of salt-soluble proteins (i.e., albumins and globulins) remained relatively constant among the eight maturation stages with values from 1.2 to 1.9% (Table 1).

**Table 1:** Protein content, composition, and characterization of developing kernels of bread wheat cv. ‘Arnold’

Trait^a^	Days after anthesis							
	7	11	14	18	25	33	39	46
PROT (g/100 g)	17.7^a^	14.7^b^	13.0^bc^	12.3^c^	13.3^bc^	13.6^bc^	13.9^bc^	14.5^b^
ALBGLO (g/100 g)	1.9^a^	1.4^bc^	1.5^bc^	1.7^ab^	1.3^bc^	1.2^c^	1.6^abc^	1.7^ab^
ATI (g/100 g)	n.d.	0.2^d^	0.4^c^	0.5^b^	0.6^b^	0.6^b^	0.7^a^	0.7^a^
TIA (mg/kg)	n.d.	n.d.	n.d.	n.d.	<LOQ	79.6^b^	89.7^a^	79.1^b^

^a^ *PROT* crude protein content by Dumas method; *ALBGLO* combined albumin and globulin content by Bradford method; *ATI* ATI content by RP-HPLC; *TIA* trypsin inhibitory activity *n.d.* not detected; *<LOQ* trypsin inhibitory activity below 40%. Means denoted by a different letter indicate significant differences between sampling dates (*p* < 0.05)

ATI proteins extracted from wheat kernels were quantified by RP-HPLC. No ATIs could be detected in the first and only marginal amounts in the second developmental stage. Hence, initial ATI accumulation occurred not before 1 week after anthesis. Subsequently, the concentration increased gradually until grain maturity and resulted in a maximum ATI concentration of 0.7 g/100 g at 46 DAA (Table 1).

### Characterization of ATIs by MALDI-TOF MS

**Fig. 3:**
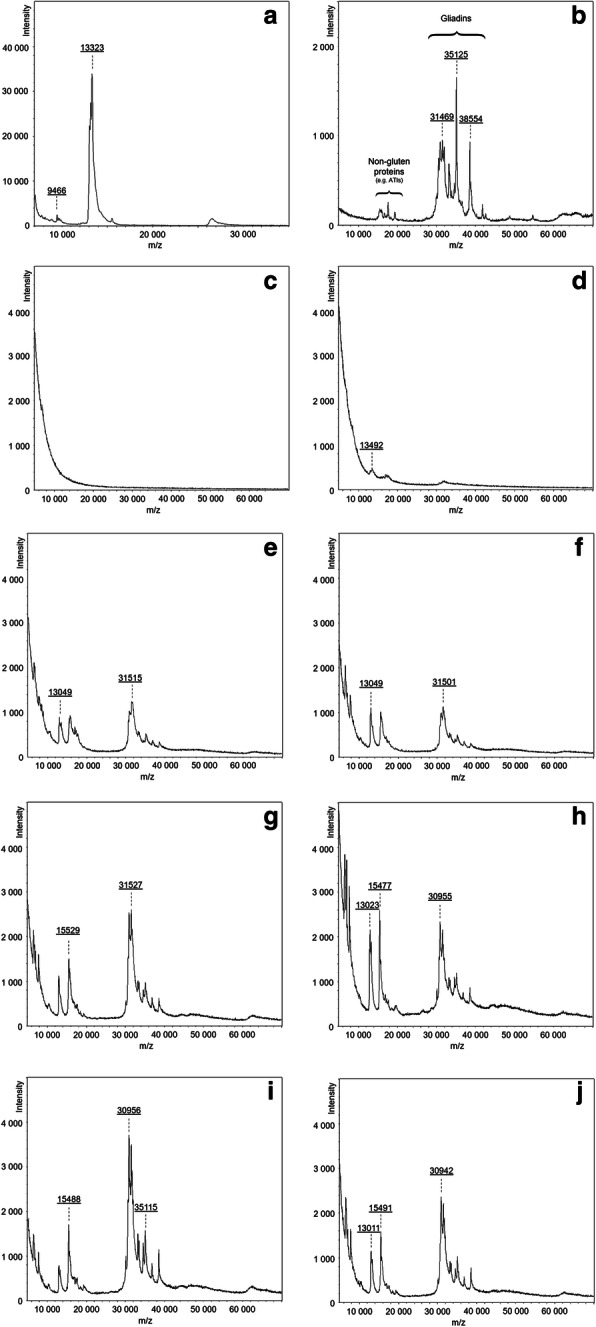
MALDI-TOF MS spectra of proteins from developing wheat kernels extracted with chloroform-methanol after SPE purification in the range of 5–70 kDa. **a** AAI standard (in the range of 7–35 kDa). **b** PWG gliadin. **c-j** ‘Arnold’ grains sampled 7, 11, 14, 18, 25, 33, 39, and 46 days after anthesis, respectively

Sample extracts were analysed by MALDI-TOF MS in order to verify the presence of ATIs. Since ATI aggregates are stabilized by rather weak non-covalent forces and thus, easily disrupted by low or high pH, denaturating or organic reagents, ATIs were mainly identified and quantified in their monomeric form. Spectra of the samples are shown in Fig. 3. The α-amylase inhibitor (AAI) standard from wheat (Fig. 3a), purchased from Sigma-Aldrich, showed a strong peak accumulation at around 12–14 kDa indicating the presence of several different AAIs and CM proteins with similar molecular weights. Furthermore, a small peak around 15.5 kDa was detected, which can be assigned as CM3 protein [19]. The broad peak at 26–27 kDa represents [2 M + H]^+^ ions of the detected ATIs. Furthermore, distinct signals with lower molecular weights (around 6 kDa and 10 kDa, respectively) were spotted in the AAI standard spectrum, which were resulting from the high amount of impurities present in the AAI standard [20, 21]. The PWG gliadin isolate (Fig. 3b) illustrated a typical gliadin pattern with a majority of α−/ß-gliadins from 30 to 40 kDa and minor abundance of ω-gliadins from 40 to 55 kDa. Besides gluten type proteins, the PWG spectrum revealed the presence of ATIs and avenin-like proteins, which was recently confirmed by Lexhaller et al. [22]. As ATIs are rather small proteins that range from 13 to 18 kDa [20, 23], sample peaks in this area were assigned predominately to ATIs and to other proteins from the albumin/globulin fraction (e.g. avenin-like proteins) in a minor extent [24]. The first sample from 7 DAA revealed a complete absence of ATIs and other storage proteins. Only an undefined and strong increase of the baseline below 10 kDa was observable, which probably resulted from intermediate peptides and free amino acids [25]. The second sample collected 4 days later showed weak peak intensities around 13, 16 and 32 kDa, which indicated the biosynthesis of the mentioned proteins. Samples taken 14 and 18 DAA presented a clear onset of ATI accumulation simultaneous to the synthesis of gliadins, which were represented by peaks > 30 kDa. Comparison of signal intensities arising from ATIs and gliadins revealed no trend for later developmental stages, which might be influenced by manual sample preparation for MALDI-TOF analysis.

### Characterisation of ATI functionality

The biological function of ATIs was described in terms of trypsin inhibitory activity (TIA) by an enzymatic assay [20]. Despite the presence of ATIs at an earlier stage, the onset of inhibitory activity occurred later as the samples taken 7 to 18 DAA showed no detectable TIA (Table 1). An initial inhibitory activity was obtained at 25 DAA, however not quantifiable as the minimum inhibition for a proper quantification (i.e. 40%) was not reached. Samples of later developmental stages showed considerable activities from 79.1 mg/kg to 89.7 mg/kg.

### Carbohydrate quantification by HPAEC-PAD

**Fig. 4:**
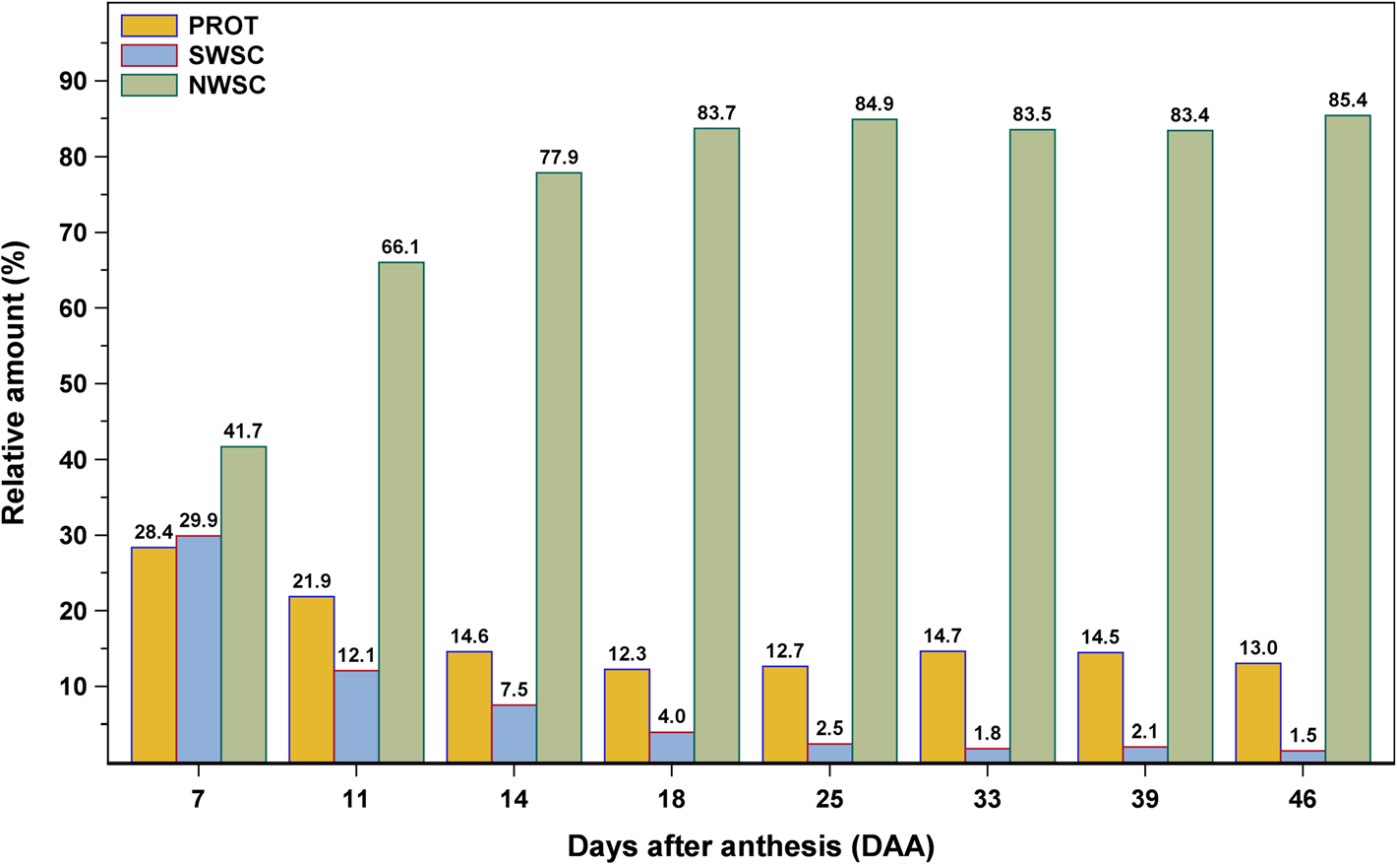
Relative distribution of the major kernel components (i.e., crude protein content (PROT), small water-soluble carbohydrates (SWSC) and non-water-soluble carbohydrates (NWSC)) during grain development of bread wheat cv. ‘Arnold’

Extraction of soluble mono-, di- and oligosaccharides from the maturing wheat kernels was performed in water without heating. In order to gain further insights into the composition of carbohydrates, the insoluble residues were subjected to a strong acid hydrolysis with 2.5 M TFA. As a result, insoluble carbohydrates were depolymerized to monomers and readily quantifiable by HPAEC-PAD. Changes in carbohydrate compositions of the samples are shown in Fig. 4 and Table 2.

**Table 2:** Compositional changes in the profile of small water-soluble carbohydrates (SWSC) and non-water-soluble carbohydrates (NWSC) in g/100 g throughout grain development

Trait^a^	Days after anthesis							
	7	11	14	18	25	33	39	46
SWSC	18.7^a^	8.1^b^	6.7^c^	4.0^d^	2.6^e^	1.7^f^	2.0^ef^	1.7^f^
GAL	0.05^a^	0.03^b^	0.03^c^	0.02^d^	0.02^d^	0.02^e^	0.01^f^	0.01^f^
GLU	4.82^a^	2.36^b^	1.70^c^	1.09^d^	0.61^e^	0.07^f^	0.05^f^	0.05^f^
FRU	8.13^a^	2.94^b^	2.05^c^	1.31^d^	0.68^de^	0.08^e^	0.05^e^	0.05^e^
SUC	0.09^c^	0.09^c^	0.09^c^	0.05^c^	0.06^c^	0.59^b^	0.72^a^	0.68^a^
RAF	0.24^c^	0.19^cd^	0.13^de^	0.08^e^	0.13^de^	0.38^ab^	0.42^a^	0.32^b^
STA	0.02^c^	0.03^b^	0.27^a^	n.d.	n.d.	n.d.	n.d.	n.d.
VER	2.50^a^	1.29^b^	1.09^c^	0.53^d^	0.07^e^	0.02^e^	0.01^e^	0.01^e^
MAL	1.49^a^	0.65^d^	0.92^b^	0.58^d^	0.76^c^	0.32^f^	0.44^e^	0.31^f^
FOS	1.33^a^	0.55^b^	0.40^c^	0.34^c^	0.28^c^	0.22^c^	0.28^c^	0.28^c^
NWSC	26.0^c^	44.4^bc^	69.1^ab^	83.7^a^	89.4^a^	77.6^a^	80.2^a^	95.3^a^
WU-GAL	0.50^a^	0.38^ab^	0.25^b^	0.37^ab^	0.43^ab^	0.55^a^	0.49^a^	0.49^a^
WU-AX	5.99^c^	8.32^abc^	11.9^ab^	12.5^a^	9.73^abc^	7.90^bc^	7.62^bc^	7.44^c^
ARA/XYL	1.04^a^	0.75^a^	0.49^a^	0.54^a^	0.60^a^	0.83^a^	0.79^a^	0.69^a^
STARCH	19.6^d^	35.7^cd^	57.0^bc^	70.8^ab^	79.3^ab^	69.1^ab^	72.1^ab^	87.3^a^

^a^*SWSC* small water-soluble carbohydrates; *GAL* galactose; *GLU* glucose; *FRU* fructose; *SUC* sucrose; *RAF* raffinose; *STA* stachyose; *VER* verbascose; *MAL* maltose; *FOS* short-chain fructooligosaccharides (sum of GF2, GF3 and GF4); *NWSC* non-water-soluble carbohydrates; *WU-GAL* water-unextractable galactose; *WU-AX* water-unextractable arabinoxylans (sum of arabinose and xylose after acid hydrolysis); *ARA/XYL* arabinose/xylose ratio; *STARCH* starch. Means denoted by a different letter indicate significant differences between sampling dates (*p* < 0.05)

#### Soluble carbohydrate profile

In general, the total amount of soluble carbohydrates was decreasing throughout grain development from 18.7 to 1.7%. Concentrations of all mono-, di- and oligosaccharides present were decreasing, except of sucrose, which was found only in traces at the beginning of grain development, but showed a final amount of 0.7%. The range of raffinose concentrations remained relatively stable over 46 days, whereas other galacto-oligosaccharides such as stachyose and verbascose could not be detected in later developmental stages or only in very small amounts. The course of changes in glucose and fructose concentrations were almost identical and were the highest during the phase of cell division and expansion with maximum values of 4.8% for glucose and 8.1% for fructose. During grain development both concentrations decreased significantly resulting in low amounts of 0.05% for glucose and fructose in the mature kernels. Sucrose concentrations were similar to glucose and fructose decreasing slightly during grain filling, but significantly increasing during grain maturation and desiccation. Due to the opposing behavior of glucose, fructose and sucrose, a shift in the monosaccharide (sum of glucose and fructose) to sucrose ratio could be observed. Besides free glucose and fructose concentrations, changes in the fructan content were found to be the most striking alteration during grain development. The amount of low molecular weight fructans (GF2-GF4) was rapidly decreasing during the milky stage (7 to 11 DAA) from an initial concentration of 1.3 to 0.6% (Table 2). After the milky stage only a minimal reduction to a final amount of 0.3% occurred.

#### Insoluble carbohydrate composition

As described by Pritchard et al. [26], the sum of arabinose and xylose monosaccharides represents the amount of arabinoxylans. In this case, water-unextractable arabinoxylan (WU-AX) content was determined, which is usually twice that of water-extractable arabinoxylans (WE-AX) in wheat flour [27]. In general, WU-AX concentrations were increasing during grain filling and maturation from 6.0 to 7.4% with a maximum of 12.5% at 18 DAA (Table 2). Arabinoxylans can be classified according to their molecular structure, which is typically expressed by the arabinose to xylose ratio (ARA/XYL). ARA/XYL of the investigated samples were found between 0.5 and 1.0. Besides WU-AX, low amounts (0.3 to 0.6%) of water-unextractable galactose were detected in the grain samples (Table 2).

Insoluble wheat starch in the grain samples was represented by the amount of glucose after acid hydrolysis as the first extraction with water should have removed free glucose and β-glucan present in the grain sample. Thus, glucose in the water-insoluble residue derived predominantly from starch. In contrast to the classical method for starch quantification, the approach used in this study might also cover other insoluble glucose-containing polysaccharides, such as cellulose, to a minor extent. Throughout grain filling and maturation starch concentrations were increasing from 19.6 to 87.3% (Table 2). While the amount was enormously increasing during the first two phases of grain development (from 19.6 to 79.3%), samples of the grain maturation and desiccation phase showed rather constant values between 69.1 and 87.3% (Fig. 2a). A similar course of accumulation was monitored on a single kernel basis (Fig. 2b). In general, the course of starch accumulation in the grain was highly correlated to the corresponding dry kernel weight. The crude protein content was also found to be negatively correlated with starch concentrations (Table 3).

**Table 3:** Pearson correlation coefficients between major kernel components and protein fractions. Probabilities of significance are indicated as superscripted values

Major compositional characteristics			
	Kernel dry weight	Crude protein content	Starch content
Kernel size	0.669^0.005^	−0.755^0.001^	0.709^0.002^
Kernel dry weight		−0.557^0.025^	0.874^0.000^
Crude protein content			−0.714^0.002^
**Protein characteristics**			
	**ALBGLO**	**ATI**	**TIA**
Crude protein content	0.436^0.092^	−0.180^0.537^	0.621^0.101^
ALBGLO^a^		0.239^0.411^	0.233^0.579^
ATI			0.612^0.107^

^a^*ALBGLO* sum of albumins and globulins; *ATI* amylase-trypsin inhibitor; *TIA* trypsin inhibition activity

## Discussion

In this study, wheat samples were collected during grain development in order to monitor the accumulation of ATIs as well as compositional changes in the carbohydrate profile. The results revealed important insights into their expression profiles during specific developmental stages.

### Kernel growth and crude protein accumulation

The determination of dry kernel weight and kernel size by Verspreet et al. [17] and Gegas et al. [28] revealed similar values for mature wheat grains as reported in this study, with a dry weight of 51.4 ± 1.8 mg/kernel [17] and a mean kernel size of 22.8 ± 3.6 mm^2^ [28], respectively.

In general, relative crude protein content, which was calculated based on the total nitrogen content in the sample, was decreasing from 17.7% at early grain filling to 14.5% in the mature kernel (Fig. 2a). A similar decline of crude protein has been reported in earlier studies on immature wheat [17, 25, 29]. Furthermore, Jennings and Morton [25] demonstrated that at the beginning of grain development about 50% of the total nitrogen was present in protein, while the remaining half was mostly present in free amino acids. However, as the amount of free amino acids and peptides was decreasing during grain development, the majority of the total nitrogen compounds were found to consist of proteins, except for kernels in the very first phase of development [25]. Protein synthesis and assembly were identified as principal functions of the wheat endosperm during grain development, with predominance in the early stages of grain development [14]. The strong MALDI-TOF MS signals between 5 and 10 kDa at early grain development which decreased thereinafter confirmed the presence of bigger peptides at the beginning of grain filling and conversion to proteins with higher masses during grain maturation. The Dumas method was designed to determine the nitrogen content by combustion and a factor (i.e. 5.7) is used to convert the nitrogen content to the crude protein content. Because of the mentioned procedure, the crude protein content includes all amino acids, peptides and proteins independently of their size.

The decrease of the total crude protein content observed in the first third of grain development might be due to the extensive NWSC accumulation, mainly starch (Fig. 4). This decrease was followed by a vast accumulation of crude protein during the grain filling and maturation phases, which was mentioned before in other studies [17, 25, 29]. The slight increase of protein in the later stages of growth might result from the final desiccation prior to maturation. Correlation analysis of total crude protein content and kernel size indicated a significant negative correlation (Table 3), as samples with kernel sizes about 25 mm^2^ showed the lowest protein concentrations (Fig. 1 and Fig. 2a). The presented values of salt-soluble proteins were ranging from 1.2 to 1.9% which comply with ALBGLO contents presented in an earlier study [19]. Albumin and globulin concentrations remained relatively stable throughout grain development (Table 1) and were not significantly correlated to crude protein contents (Table 3). In general, contents of total protein and salt-soluble proteins of grains from the final maturity were similar to those of cv. ‘Arnold’ from the year 2017 and 2018 as presented by Call et al. [19]. The results on kernel growth and protein accumulation were in agreement with general knowledge on grain development.

### ATI accumulation and trypsin inhibition

As shown in Table 1, ATI content increased steadily from 7 DAA until final maturity. These findings were in accordance with Finnie et al. [13], who described the gradual accumulation of ATIs throughout barley seed development. This pattern of appearance might be attributed to their functional role in the defensive system of the cereal plant. As their role is to defend the starch and protein reserves of the seed, their accumulation is expected to be simultaneous with grain filling. Using 2D gel electrophoresis-mass spectrometry of wheat proteins also Vensel et al. [14] proved the dominance of proteins involved in stress and defence, including amylase and trypsin inhibitors, at later stages of grain development. The detected amounts of ATIs were in accordance, however slightly lower when compared to values obtained for the same wheat variety harvested in 2017 and 2018, which were 1.3 and 1.1 g/100 g, respectively [19]. The slight decrease in ATI concentrations might be due to the replacement of soy trypsin inhibitor (TI) as calibration standard to BSA. Generally, the simple RP-HPLC method used for ATI quantification is known to overestimate ATI amounts, since avenin-like proteins were co-eluting and thus, included in these results [19].

MALDI spectra of the ‘Arnold’ samples confirmed the results obtained by RP-HPLC since ATIs were not detected at 7 DAA and only marginal signals were detectable after 11 DAA. The sample taken 14 DAA showed a clear onset of ATI accumulation simultaneous to the synthesis of gliadins. These findings are in contrast to Carbonero et al. [1], who claimed that ATI synthesis precedes that of the storage proteins. However, quantity of storage proteins has not been determined in the present study.

Although ATIs could be detected at earlier stages by MALDI-TOF MS, quantification by RP-HPLC demonstrated that the samples shortly after anthesis contained rather low concentrations. Nevertheless, a certain time lag from initial ATI occurrence until biological activity of ATIs was evident throughout grain development. While the samples taken 25 and 33 DAA exhibited similar ATI concentrations (0.6 g/100 g), they significantly differ in their trypsin inhibitory potential (<LOQ and 79.6 mg/kg, respectively). Based on this discrepancy, no correlation could be observed for ATI concentration and their corresponding trypsin inhibition (Table 3) as it was also demonstrated in earlier studies [19]. The time lag that occurred might be due to the preceding assembling of ATIs to dimers and tetramers, which seems to be crucial for biological functions [1]. Based on the acquired results, the formation of biological activity of ATIs could be assigned to a maturation period between 18 and 33 DAA. In order to further narrow down this period, sample collection could be performed more frequently in follow-up studies. In general, trypsin inhibitory potential at later developmental stages were closely to results of Call et al. [19] with 66.2–84.6 mg/kg and Call et al. [20] with 73.2 mg/kg for cv. ‘Arnold’. In comparison with TIA values from other varieties, cv. ‘Arnold’ is characterized by a medium trypsin inhibitory activity [19, 20].

### Compositional changes in the carbohydrate profile

The levels of soluble sugars found in the mature grains of the present study were comparable to results from Call et al. [30], whose study found 0.03% glucose, 0.07% fructose, 0.8% sucrose and 0.3% raffinose among 19 winter bread wheat cultivars grown and harvested 2016 in Austria. In general, carbohydrate metabolism is known to be an abundant process throughout grain development [14]. The decrease of soluble mono- and disaccharides during kernel ripening reflected their progressive conversion into storage polysaccharides, as in fact, starch concentrations were increasing from 19.6 to 87.3% (Table 2).

During grain development the amount of short-chain fructans was reduced by approximately 80%. The shown levels in mature grains were in accordance with results from Peukert et al. [18], who demonstrated degradations during barley grain development from 0.26 to 0.01% for kestose (GF2) and from 0.18 to 0.16% for nystose (GF3). Probably, fructans with higher molecular weights were generated later, which was responsible for this reduction. Fraberger et al. [31] reported degrees of polymerization (DP) of up to 15 in whole meal flour of the same wheat cv. ‘Arnold’. Fructans are known to be abundant in immature wheat kernels and similar reduction rates of 50–90% have been reported for mature wheat when compared to immature kernels [16]. However, it has to be pointed out that in the present study only low molecular weight fructans (GF2-GF4) have been determined. Fructans have received much attention in the scientific literature thanks to their health benefits and physiochemical properties. Due to the high levels of fructans in immature wheat kernels, the employment of wheat grains harvested at the milk stage for the production of functional foods has been discussed [32, 33]. However, these products might then again not be suitable for people suffering from irritable bowel syndrome (IBS) as fructans are suspected to trigger symptoms of IBS [11].

Like the study conducted by Verspreet et al. [17], AX concentrations increased rapidly during the cell division and expansion phase, remained relatively stable during grain filling and resulted in slightly decreased, however stable AX amounts in the range of 7.4–7.9% in the maturation and desiccation phase (Table 2). Comparable results for WU-AX in mature grains were obtained by Cetiner et al. [34] with a mean value of 7.2% for modern wheat varieties. ARA/XYL in the present study was slightly increased when compared to typical average values of 0.5–0.6 as reported by Cleemput et al. [35]. However, ARA/XYL seemed to be increased in WU-AX due to more intensive branching [36]. Furthermore, arabinose from arabinogalactan was not considered in the quantification which might lead to adulterated ARA/XYL. AX are known to be the major component of wheat cell walls, which is reflected by the high amounts found in this study. The poor concentrations of water-unextractable galactose (WU-GAL) found in the present study could be traced back to galacto-oligosaccharides that are embedded in minor amounts in the cell wall structure as also shown by Mares and Stone [37].

In the literature, starch content was found to rarely exceed 72% of wheat dry matter [38]. The increased amounts found in this study might be caused by an incomplete β-glucan or cellulose removal due to the single cold water extraction step applied. However, the total sum of protein and carbohydrate components does not exceed 110% of the dry kernel weight, which is acceptable considering the measurement uncertainties of the numerous analytical traits that were determined for each caryopsis sample.

## Conclusions

The results provide insights into the complex compositional changes during grain filling and maturation, with particular emphasis on the protein and ATI content, as well as in the inhibition activities of ATIs towards trypsin. In general, ATI concentration and activity were found to increase throughout grain development, while fructans were abundant in immature wheat kernels. Although the accumulation of ATI monomers started in the first phase of grain development, biological activity in terms of trypsin inhibition could only be detected after a time lag of around 3 weeks. This result supports the assumption that biological function of ATIs is strongly dependent on their aggregation state and conformation. However, it has to be mentioned that the immunogenic potential of ATIs is primarily based on their activation of specific pro-inflammatory receptors. Changes in environmental conditions during grain development could additionally alter the rate of ATI synthesis and ultimately affect the immunogenic potential of the grain. Although several studies helped to understand the complex gene network that regulates protein expression during grain development, the biochemical mechanisms for ATI synthesis still require further research. The complexity of trypsin inhibition was impressively demonstrated in a recent research study: although total ATI concentrations were reduced by gene silencing of three major ATI genes, TIA was increased [39].

## Methods

### Plant materials

Bread wheat (*Triticum aestivum* L., 2n = 6x = 42, BBAADD, cv. ‘Arnold’) kernels were collected in 2019 from a field trial in Tulln an der Donau, Austria (48°18′42.2“ N, 16°03’06.8” E) at eight selected intervals over a period of 7 weeks during the grain filling and maturation stage. No permission was required to collect the plant samples. Fertilization (i.e. 67.5 kg N ha^− 1^ on 21 March and 43.2 kg N ha^− 1^ on 21 May) and plant protection (i.e. herbicide treatment with 1.35 l ha^− 1^ diflufenican on 8 April and 25 g ha^− 1^ tribenuron-methyl + 0.25 l ha^− 1^ arylex and fluroxypyr on 27 May) were performed to ensure optimal plant growth. Anthesis took place on 31 May, and hand collecting of the plants started 7 days after anthesis (DAA). For each sample at least 50 fertile tillers were cut and frozen with liquid nitrogen to inhibit further reactions or enzymatic activity. Frozen kernels were further lyophilized by a FreeZone 6 freeze dryer (Labconco, Kansas City, MO, USA) prior to analysis. Due to this drying process all data obtained in this study are based on the dry weight of the kernels. Details of the sampling of caryopses and the environmental conditions are presented in Additional file 5: Table S3. Unfertilized ovaries were excluded from analysis.

### Determination of dry kernel weight and kernel size

At least 20 freeze-dried kernels were weighted in order to determine the average kernel weight for each developmental stage. Determination of kernel size of the maturing kernel samples was conducted by digital image analysis using ImageJ2 software [40]. Calibration with a simultaneous evaluation of a defined length scale and an ellipsoid model were chosen for proper kernel size quantification. The reported values were mean values ± SEM obtained from the measurement of at least 20 kernels.

### Sample preparation for protein determination

The caryopses for analyses were manually collected from the spikes and ground under liquid nitrogen using a CryoMill (Retsch GmbH, Haan, Germany), lyophilized and stored at − 18 °C prior to analysis. For ATI determinations, 1 g of wholemeal flour was extracted twice with 10 mL *n-*hexane in order to remove sample fat and chlorophyll, especially from rather immature samples, which might interact with spectrophotometric measurements. Subsequently, ATIs and other salt-soluble proteins were extracted with 150 mM NaCl solution containing 1.3 mM phosphate buffer (pH 7). After vortexing the suspension for 20 s, the extraction was completed under vigorous shaking in an overhead shaker for 10 min. Non-soluble particles were separated by centrifugation (3000 g, 10 min) before combining the resulting supernatants. Sample preparation was performed twice for each sample.

### Determination of protein content

Crude protein content of the milled wholemeal flours was determined as described by Call et al. [19] using a DuMaster D-480 (Büchi Labortechnik AG, Flawil, Switzerland). Additionally, the amount of ALBGLO was determined using the sodium chloride extracts with a Roti®-Quant assay (Carl Roth, Karlsruhe, Germany) according to Call et al. [20].

### Quantification of ATIs by RP-HPLC

Quantification of ATIs in the extracts was performed as described in a previous study [19] with some changes in the chromatographic procedure (for details see Additional file 6: Table S4). Briefly, the prepared ATI extracts were injected on a Hitachi HPLC system from 5000 series equipped with a DAD detector, and a HALO C18 column (Advanced Materials Technology, Inc., Wilmington, DE, USA) with 1000 Å pore size (150 × 2.1 mm with 2.7 μm particle size) including a corresponding guard column. A gradient with water (solvent A) and HPLC-grade acetonitrile (ACN) (solvent B), both modified with 0.1% trifluoroacetic acid (TFA), was applied at 45 °C with a flow rate of 0.35 mL min^− 1^. Detection was performed at 214 nm. BSA with a purity of at least 98% (Carl Roth) was chosen to establish an external calibration as it is also used for the quantification of ALBGLO in the sample extracts. The applied analytical procedure will quantify the majority of ATIs, but some other proteins as well. Since no protein identification was performed, a detailed number of ATIs or list of proteins determined within assigned retention times cannot be provided.

### ATI characterization

Sample extracts containing ATIs and other salt-soluble proteins were analyzed for their trypsin inhibitory activity (TIA) according to Call et al. [20]. Samples that showed low inhibition but did not reach minimum inhibition of 40% were declared as lower LOQ. TIA values were expressed in mg inhibited trypsin per kg sample.

Samples of different developmental stages were further characterized by MALDI-TOF MS according to Call et al. [20] with some modifications. Proteins were extracted from defatted wholemeal flour with a chloroform-methanol solution (equal ratio). The obtained extracts were evaporated at 50 °C under nitrogen stream to dryness and resuspended in an acetonitrile-water solution (equal ratio) containing 0.1% TFA. Purification and enrichment of proteins was performed with C4 ZipTips (0.6 μL bed volume, 10 μL total pipette volume) purchased from Merck according to instructions of the manufacturer.

### Carbohydrate analysis

In order to determine changes in the carbohydrate metabolism throughout grain development, 50 to 100 mg of sample were extracted in 5 mL water for 10 min in an overhead shaker. Insoluble components such as starch and precipitated proteins were removed by centrifugation (3000 g, 10 min). The extracts were filtered through a 0.2 μm filter prior to chromatographic analysis. To gain further information on the insoluble carbohydrate components, the insoluble residue after centrifugation was hydrolysed by 5 mL 2.5 M TFA in a shaking water bath at 100 °C for 2 h. As previously described by Fraberger et al. [31], proteins were removed by Carrez precipitation and subsequent centrifugation. Samples extracts were adjusted to a pH > 6 by adding NaOH, filtered and injected into the chromatographic system.

To quantify carbohydrate concentrations in the sample extracts, HPAEC-PAD was applied according to Fraberger et al. [31]. A Dionex™ ICS-6000 DC system (ThermoFisher Scientific, Sunnyvale, CA, USA) equipped with a CarboPac™ PA210 (2 × 150 mm, 4 μm) and a CarboPac™ PA20 Fast (2 × 100 mm, 4 μm) with corresponding guard columns was used for the separation of different carbohydrates. A gradient elution with 200 mM NaOH and 200 mM NaOH/500 mM Na-acetate was used at a flow rate of 0.2 mL/min and at a column temperature of 30 °C. Details of the chromatographic conditions are shown in Additional file 8: Table S5. The calibration for the method was performed with galactose, glucose, fructose, sucrose, raffinose, maltose and smaller fructans from DP 3–5 (Megazyme, Bray, Ireland) in a range from 0.5 to 50 mg/L.

### Collection of environmental data

Climatic data for the test site were collected by a A733 telemetry unit (ADCON, Klosterneuburg, Austria) equipped with sensors for temperature, humidity, wind, radiation and precipitation. Temperature and precipitation of the growing season (19 October 2018–16 July 2019) are presented in Additional file 9: Fig. S4.

### Statistical analysis

Samples were extracted at least twice and analysed one to two times depending on the reproducibility of the respective trait measurement which was determined in previous experiments. All statistical analyses were performed with SAS 9.4 software (SAS Institute, Inc., Cary, NC). Procedure MIXED was used for analysis of variance with DAA as fixed effect. Mean comparisons were calculated using the Tukey-Kramer method. Differences with *p*-values < 0.05 were considered significant. Procedure CORR was applied to calculate correlations between traits. Procedure TEMPLATE was used to create the statistical graphs.

## Supplementary Information

**Additional file 1 Fig. S1:** Changes of grain characteristics during seed development of bread wheat cv. ‘Arnold’. **a** Grain size (*n* > 20) and **b** grain weight (*n* > 20).

**Additional file 2 Fig. S2** RP-HPLC chromatograms (214 nm) of salt-water extracts from developing kernels of bread wheat cv. ‘Arnold’. **a-h** Grains harvested at 7, 11, 14, 18, 25, 33, 39, and 46 days after anthesis, respectively.

**Additional file 3 Table S1.** Protein content, composition and characterization of developing grains of bread wheat (*Triticum aestivum* L.) cv. ‘Arnold’ on a single kernel basis.

**Additional file 4 Table S2.** Compositional changes in the SWSC (small water-soluble carbohydrates) and NWSC (non-water-soluble carbohydrates) profile throughout grain development of bread wheat cv. ‘Arnold’ on a single kernel basis.

**Additional file 5 Table S3.** Sample details including time of harvest and environmental data from anthesis until harvest.

**Additional file 6 Table S4.** Chromatographic conditions for ATI quantification by RP-HPLC.

**Additional file 7 Fig. S3.** RP-HPLC chromatogram (214 nm) of the wheat AAI standard.

**Additional file 8 Table S5.** Chromatographic conditions for carbohydrate analysis by HPAEC-PAD.

**Additional file 9 Fig. S4.** Climatic data for the test site Tulln an der Donau for the growing season from 19 October 2018 to 16 July 2019. Blue bars represent the total precipitation per month; the solid red line and respective values in the graph represent the mean monthly temperature, while minimum and maximum daily temperature means are represented by the light red band.

## Data Availability

Additional data generated or analysed during this study that are not included in this article or its additional files are available upon request from the corresponding author.

## Abbreviations

AAI: Alpha-amylase inhibitor
ALBGLO: Albumin plus globulin content
ARA/XYL: Arabinose/xylose ratio
ATIs: Amylase-trypsin inhibitors
AX: Arabinoxylans
BBCH: Biologische Bundesanstalt für Land- und Forstwirtschaft, Bundessortenamt und Chemische Industrie
BSA: Bovine serum albumin
DAA: Days after anthesis
DP: Degree of polymerization
FODMAPs: Fermentable oligo-, di-, monosaccharides and polyols
FOS: Short-chain fructooligosaccharides
FRU: Fructose
GAL: Galactose
GF_x_: Number of glucose and fructose units
GLU: Glucose
IBS: Irritable bowel syndrome
LOQ: Limit of quantification
MAL: Maltose
NCWS: Non-celiac wheat sensitivity
NWSC: Non-water-soluble carbohydrates
PROT: Crude protein content
RAF: Raffinose
SEM: Standard error of the mean
STA: Stachyose
SUC: Sucrose
TFA: Trifluoroacetic acid
TIA: Trypsin inhibitory activity
TLR4: Toll-like receptor 4
TSN: Total soluble nitrogen
SWSC: Small water-soluble carbohydrates
VER: Verbascose
WU-AX: Water-unextractable arabinoxylans
WU-GAL: Water-unextractable galactose
