# Supplementary figures and images for: Synthesis and accumulation of amylase-trypsin inhibitors and changes in carbohydrate profile during grain development of bread wheat (Triticum aestivum L.)
Source: BMC Plant Biol. 2021 Feb 24;21:113. doi: 10.1186/s12870-021-02886-x (PMC7905651; doi:10.1186/s12870-021-02886-x)

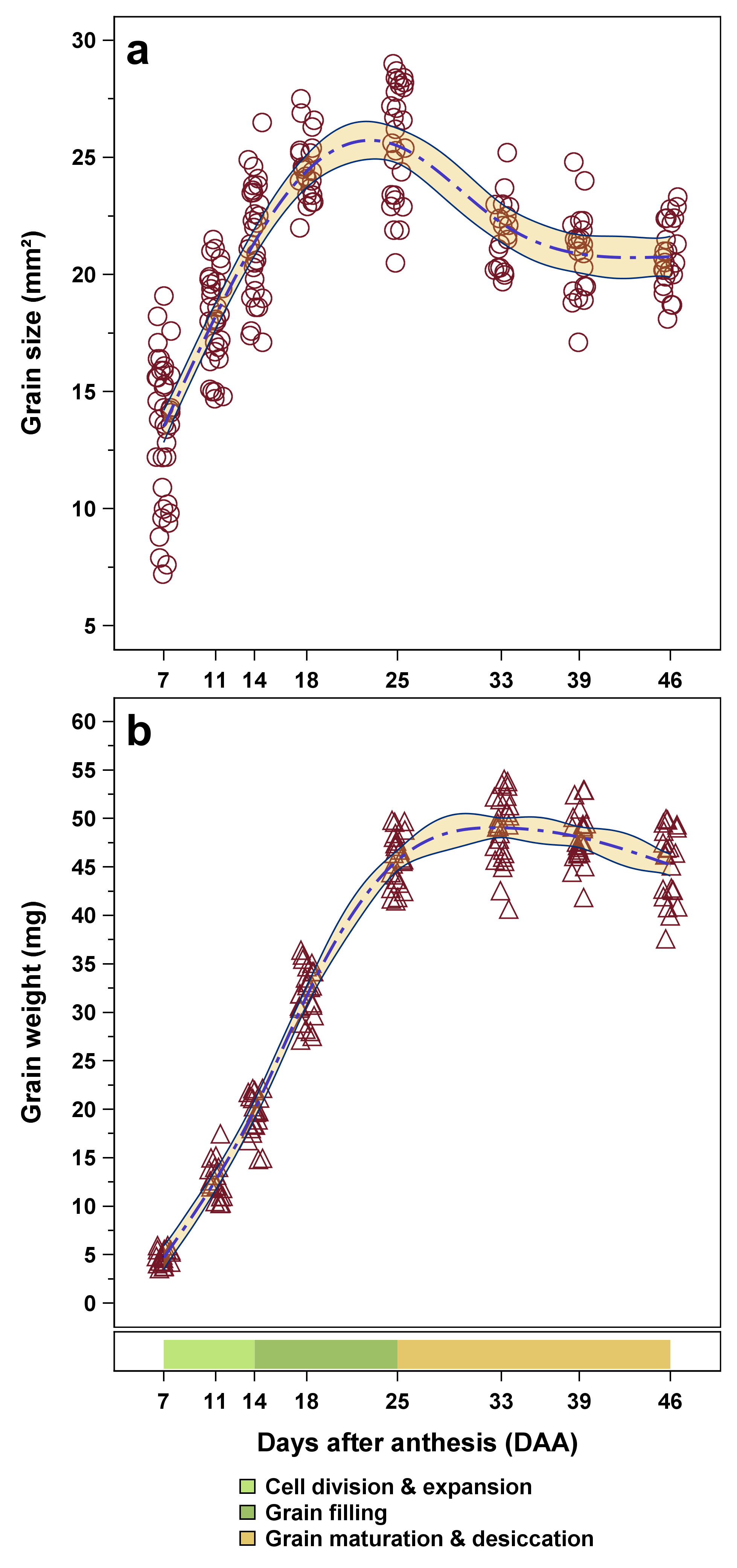

Supplement: Supplementary file 1 — Additional file 1 Fig. S1: Changes of grain characteristics during seed development of bread wheat cv. ‘Arnold’. a Grain size (n > 20) and b grain weight (n > 20). [file 12870_2021_2886_MOESM1_ESM.png]

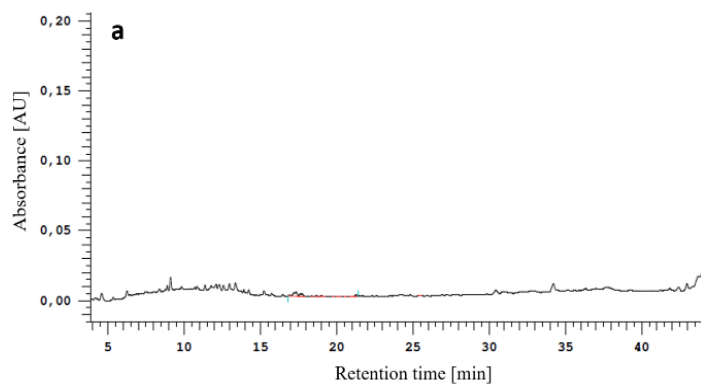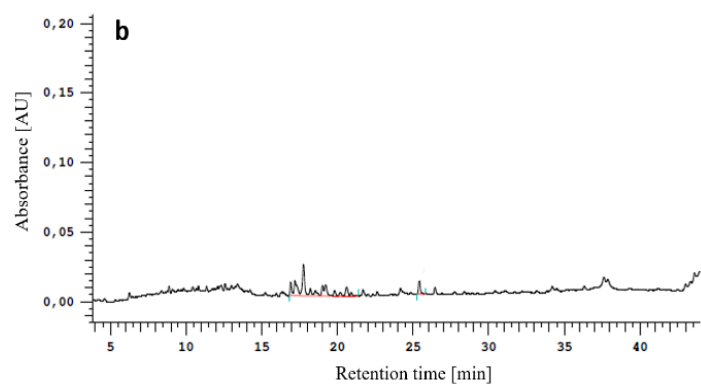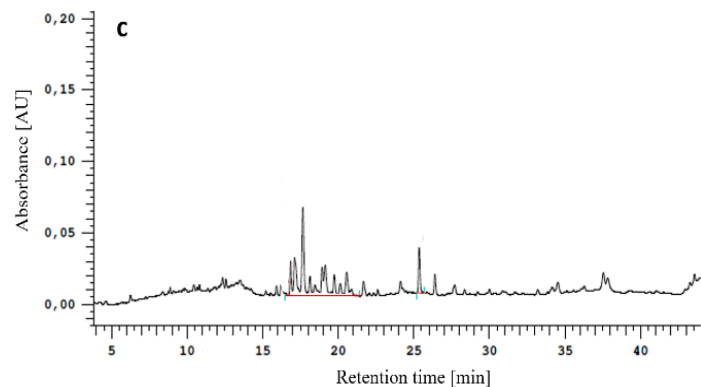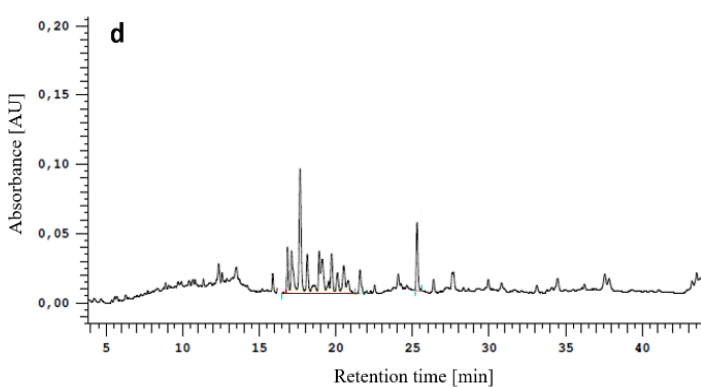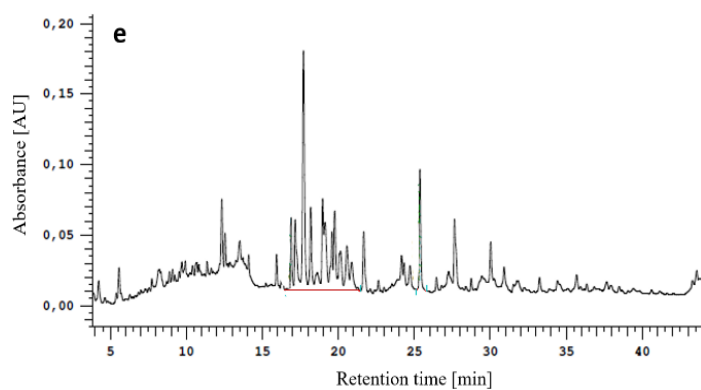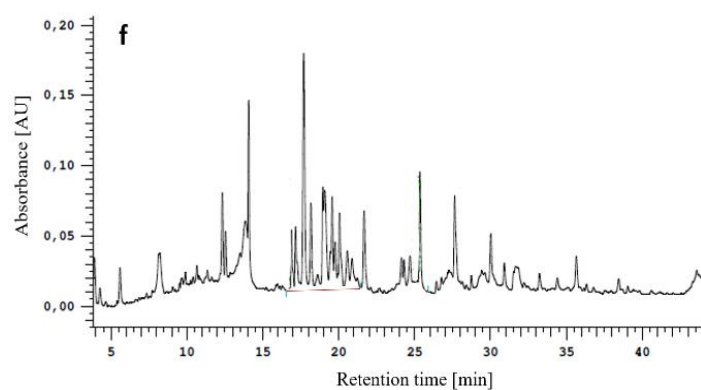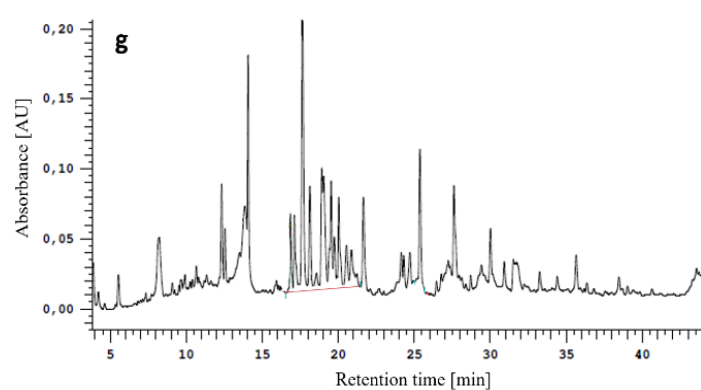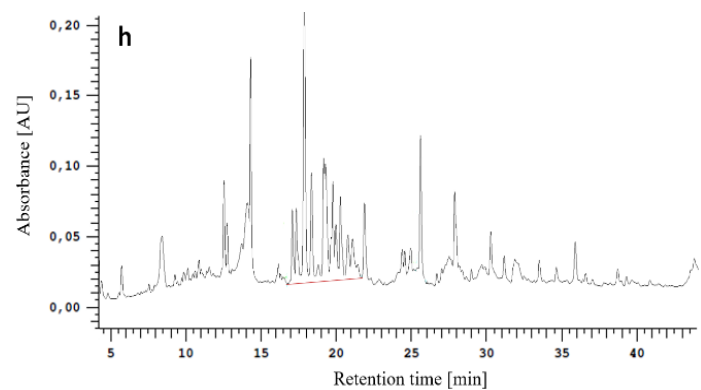

Supplement: Supplementary file 2 — Additional file 2 Fig. S2 RP-HPLC chromatograms (214 nm) of salt-water extracts from developing kernels of bread wheat cv. ‘Arnold’. a-h Grains harvested at 7, 11, 14, 18, 25, 33, 39, and 46 days after anthesis, respectively. [file 12870_2021_2886_MOESM2_ESM.pdf]

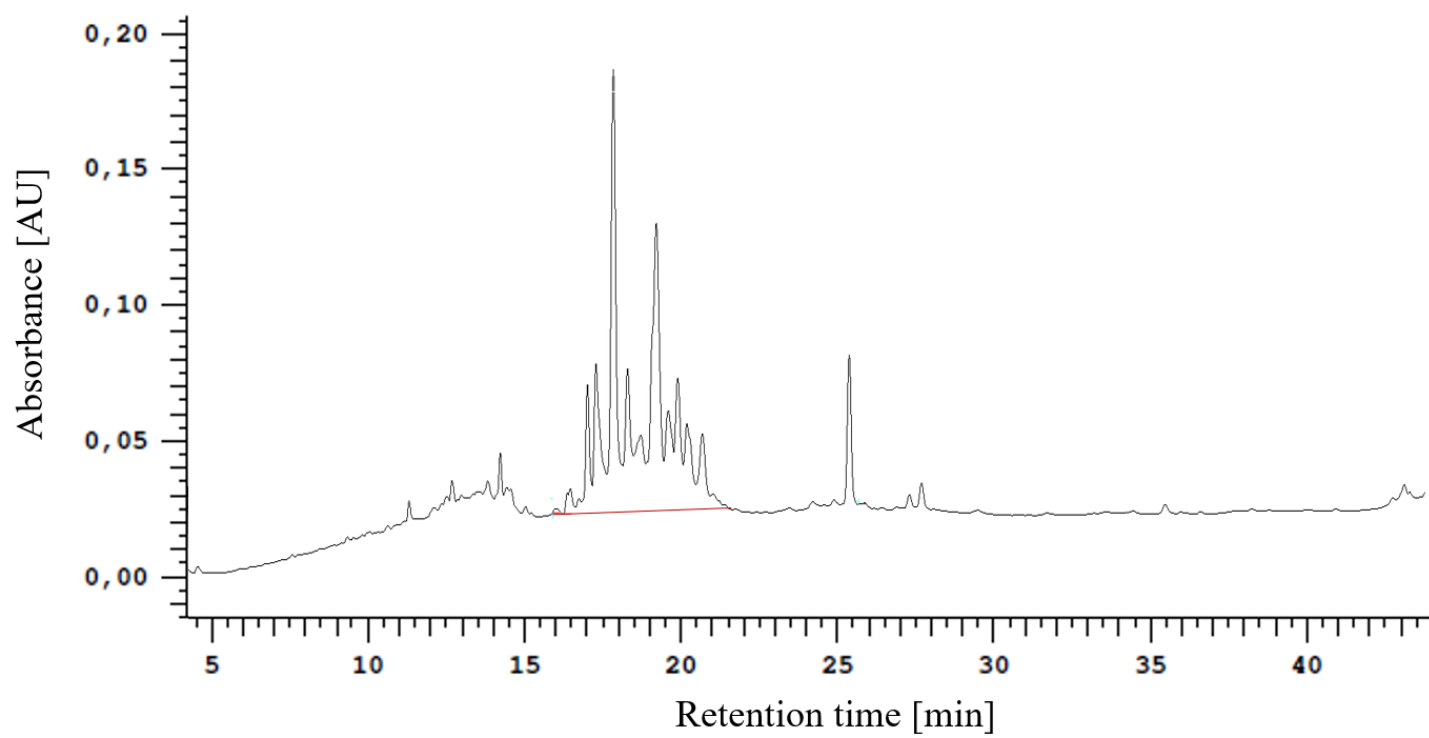

Supplement: Supplementary file 7 — Additional file 7 Fig. S3. RP-HPLC chromatogram (214 nm) of the wheat AAI standard. [file 12870_2021_2886_MOESM7_ESM.pdf]

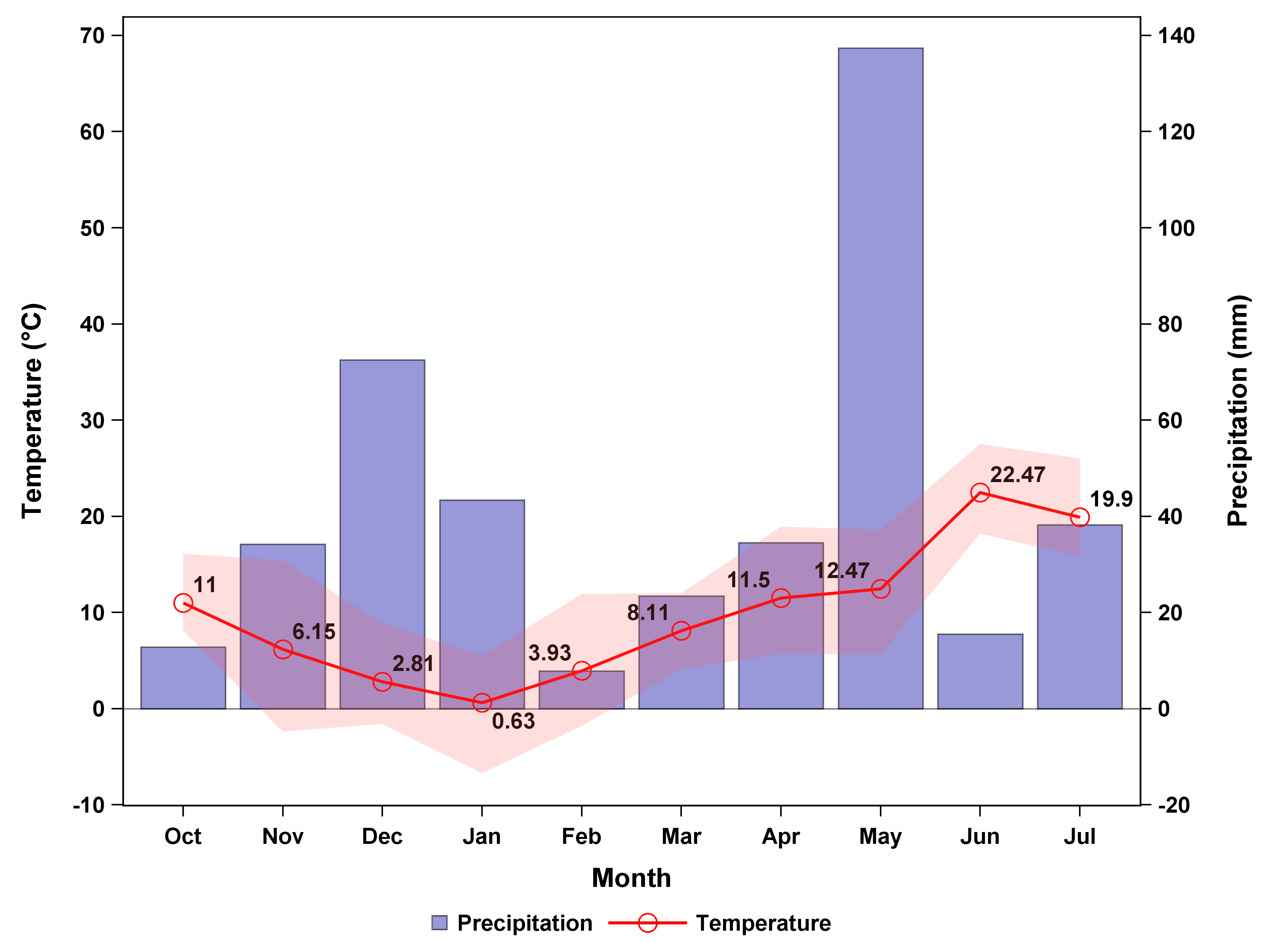

Supplement: Supplementary file 9 — Additional file 9 Fig. S4. Climatic data for the test site Tulln an der Donau for the growing season from 19 October 2018 to 16 July 2019. Blue bars represent the total precipitation per month; the solid red line and respective values in the graph represent the mean monthly temperature, while minimum and maximum daily temperature means are represented by the light red band. [file 12870_2021_2886_MOESM9_ESM.png]
