# Supplementary material for: Synthesis and accumulation of amylase-trypsin inhibitors and changes in carbohydrate profile during grain development of bread wheat (Triticum aestivum L.)
Source: BMC Plant Biol. 2021 Feb 24;21:113. doi: 10.1186/s12870-021-02886-x (PMC7905651; doi:10.1186/s12870-021-02886-x)
Supplement: Supplementary file 3 — Additional file 3 Table S1. Protein content, composition and characterization of developing grains of bread wheat (Triticum aestivum L.) cv. ‘Arnold’ on a single kernel basis. [file 12870_2021_2886_MOESM3_ESM.pdf]

Table S1 Protein content, composition and characterization of developing kernels of bread wheat cv. ‘Arnold’ on a single kernel basis.

Trait[1]	Days after anthesis							
	7	11	14	18	25	33	39	46
PROT (mg/kernel)	0.9[f]	1.9[e]	2.5[d]	3.9[c]	6.1[b]	6.7[a]	6.7[a]	6.6[a]
ALBGLO (mg/kernel)	0.1[d]	0.2[d]	0.3[d]	0.5[c]	0.6[bc]	0.6[c]	0.8[a]	0.7[ab]
ATI (mg/kernel)	n.d.	0.02[e]	0.08[d]	0.18[c]	0.26[b]	0.29[ab]	0.34[a]	0.32[a]
TIA (ng/kernel)	n.d.	n.d.	n.d.	n.d.	<LOQ	39.0[b]	43.1[a]	35.8[c]

[1] PROT, crude protein content by Dumas method; ALBGLO, combined albumin and globulin content by Bradford method; ATI, ATI content by RP-HPLC; TIA, trypsin inhibitory activity; n.d., not detected; <LOQ, trypsin inhibitory activity below 40%. Means denoted by a different letter indicate significant differences between treatments ($p < 0.05$).
