# Supplementary material for: Synthesis and accumulation of amylase-trypsin inhibitors and changes in carbohydrate profile during grain development of bread wheat (Triticum aestivum L.)
Source: BMC Plant Biol. 2021 Feb 24;21:113. doi: 10.1186/s12870-021-02886-x (PMC7905651; doi:10.1186/s12870-021-02886-x)
Supplement: Supplementary file 4 — Additional file 4 Table S2. Compositional changes in the SWSC (small water-soluble carbohydrates) and NWSC (non-water-soluble carbohydrates) profile throughout grain development of bread wheat cv. ‘Arnold’ on a single kernel basis. [file 12870_2021_2886_MOESM4_ESM.pdf]

Table S2 Compositional changes in the profile of small water-soluble carbohydrates (SWSC) and non-water-soluble carbohydrates (NWSC) in mg/kernel throughout grain development of bread wheat cv. 'Arnold' on a single kernel basis.

Trait[1]	Days after anthesis							
	7	11	14	18	25	33	39	46
SWSC	0.9^{bc}	1.0^{b}	1.3^{a}	1.3^{a}	1.2^{a}	0.8^{cd}	1.0^{bc}	0.8^{d}
GAL	0.002^{g}	0.004^{f}	0.005^{e}	0.007^{c}	0.01^{a}	$0.009b^{e}$	0.007^{cd}	0.006^{de}
GLU	0.2^{d}	0.3^{bc}	0.3^{ab}	0.3^{a}	0.3^{c}	0.04^{e}	0.02^{e}	0.02^{e}
FRU	0.4^{a}	0.4^{a}	0.4^{a}	0.4^{a}	0.3^{b}	0.04^{c}	0.02^{c}	0.02^{c}
SUC	0.005^{c}	0.01^{c}	0.02^{c}	0.02^{c}	0.03^{c}	0.3^{b}	0.3^{a}	0.3^{a}
RAF	0.01^{d}	0.02^{d}	0.03^{d}	0.03^{d}	0.06^{c}	0.2^{c}	0.2^{c}	0.1^{b}
STA	0.001^{c}	0.004^{b}	0.05^{a}	n.d.	n.d.	n.d.	n.d.	n.d.
VER	0.1^{c}	0.2^{b}	0.2^{a}	0.2^{b}	0.03^{d}	0.01^{e}	0.004^{e}	0.006^{e}
MAL	0.07^{e}	0.08^{e}	0.2^{c}	0.2^{bc}	0.3^{a}	0.2^{cd}	0.2^{b}	0.1^{d}
FOS	0.06^{c}	0.07^{c}	0.08^{c}	0.1^{ab}	0.1^{ab}	0.1^{b}	0.1^{a}	0.1^{ab}
NWSC	1.3^{a}	5.6^{ab}	13.4^{b}	26.8^{c}	40.9^{d}	38.0^{d}	38.6^{d}	43.0^{d}
WU-GAL	0.02^{d}	0.05^{d}	0.05^{d}	0.1^{c}	0.2^{b}	0.3^{a}	0.2^{ab}	0.2^{ab}
WU-AX	0.3^{d}	1.1^{cd}	2.3^{bc}	4.0^{a}	4.5^{a}	3.9^{a}	3.7^{a}	3.4^{ab}
ARA/XYL	0.05^{e}	0.1^{e}	0.1^{e}	0.2^{d}	0.3^{c}	0.4^{a}	0.4^{ab}	0.3^{bc}
STARCH	0.9^{d}	4.5^{cd}	11.1^{c}	22.6^{b}	36.3^{a}	33.8^{a}	34.7^{a}	39.5^{a}

[1] SWSC, small water-soluble carbohydrates; GAL, galactose; GLU, glucose; FRU, fructose; SUC, sucrose; RAF, raffinose; STA, stachyose; VER, verbascose; MAL, maltose; FOS, short-chain fructooligosaccharides (sum of GF2, GF3 and GF4); NWSC, non-water-soluble carbohydrates; WU-GAL, water-unextractable galactose; WU-AX, water-unextractable arabinoxylans (sum of arabinose and xylose after acid hydrolysis); ARA/XYL, arabinose/xylose ratio; STARCH, starch. Means denoted by a different letter indicate significant differences between treatments ($p<0.05$).
