# Supplementary material for: Synthesis and accumulation of amylase-trypsin inhibitors and changes in carbohydrate profile during grain development of bread wheat (Triticum aestivum L.)
Source: BMC Plant Biol. 2021 Feb 24;21:113. doi: 10.1186/s12870-021-02886-x (PMC7905651; doi:10.1186/s12870-021-02886-x)
Supplement: Supplementary file 5 — Additional file 5 Table S3. Sample details including time of harvest and environmental data from anthesis until harvest. [file 12870_2021_2886_MOESM5_ESM.pdf]

Table S3 Sample details including time of harvest and environmental data from anthesis until harvest.

Trait[1]	Days after anthesis							
	7	11	14	18	25	33	39	46
Date	7 June	11 June	14 June	18 June	25 June	3 July	9 July	16 July
ST (°C)	145.0	236.8	311.3	401.4	552.1	743.2	868.8	992.6
SP (mm)	128.2	209.5	276.7	366.2	524.2	719.3	859.7	1003.6

[1] ST: sum of temperature; SP: sum of precipitation
