# Supplementary material for: Synthesis and accumulation of amylase-trypsin inhibitors and changes in carbohydrate profile during grain development of bread wheat (Triticum aestivum L.)
Source: BMC Plant Biol. 2021 Feb 24;21:113. doi: 10.1186/s12870-021-02886-x (PMC7905651; doi:10.1186/s12870-021-02886-x)
Supplement: Supplementary file 6 — Additional file 6 Table S4. Chromatographic conditions for ATI quantification by RP-HPLC. [file 12870_2021_2886_MOESM6_ESM.pdf]

Table S4 Chromatographic conditions for ATI quantification by RP-HPLC.

Parameter	Condition
Injection volume	10 µL
Run time	62 min
Flow rate	0.35 mL/min
Column temperature	45°C
Sample loop	100 µL
Detection	214 nm
Calibration standard	Bovine serum albumin (BSA) Fraction V (0.1–1.0 g/L)
Gradient	0 min: 10% B
	10 min: 30% B
	40 min: 50% B
	45-50 min: 90% B
	51-62 min: 10% B
