# Supplementary material for: Synthesis and accumulation of amylase-trypsin inhibitors and changes in carbohydrate profile during grain development of bread wheat (Triticum aestivum L.)
Source: BMC Plant Biol. 2021 Feb 24;21:113. doi: 10.1186/s12870-021-02886-x (PMC7905651; doi:10.1186/s12870-021-02886-x)
Supplement: Supplementary file 8 — Additional file 8 Table S5. Chromatographic conditions for carbohydrate analysis by HPAEC-PAD. [file 12870_2021_2886_MOESM8_ESM.pdf]

Table S5 Chromatographic conditions for carbohydrate analysis by HPAEC-PAD.

Parameter	Condition	
	Aqueous extracts	Hydrolysed samples
Column	PA210	PA20
Column temperature	30°C	30°C
Flow rate	0.2 mL/min	0.2 mL/min
Eluent A	H_2O	H_2O
Eluent B	200 mM NaOH	200 mM NaOH
Eluent C	200 mM NaOH + 500 mM NaAc	
Calibration standards (0.5–25 mg/L)	Galactose, glucose, sucrose, fructose, raffinose, stachyose, GF2, verbascose, maltose, GF3, GF4	Arabinose, galactose, glucose, xylose, fructose
Gradient	0-10 min: isocratic (4% B)<br>10-20 min: linear (4-100% B)<br>20-21 min: isocratic (100% B)<br>21-28 min: linear (100% B-100% C)<br>28-32 min: isocratic (100% C)<br>32-32.1 min: linear (100% C-100% B)<br>32.1-36 min: isocratic (100% B)<br>36-36.1 min: linear (100-4% B)<br>36.1-52 min: isocratic (4% B)	0-7.5 min: isocratic (4% B)<br>7.5-12.5 min: linear (4-25% B)<br>12.5-20 min: linear (25-35% B)<br>20-30 min: linear (35-100% B)<br>30-45 min: isocratic (100% B)<br>45-45.1 min: linear (100-4% B)<br>45.1-60 min: isocratic (4% B)
